# Denosumab in Giant Cell Tumor of Bone: Multidisciplinary Medical Management Based on Pathophysiological Mechanisms and Real-World Evidence

**DOI:** 10.3390/cancers14092290

**Published:** 2022-05-04

**Authors:** Aneta Maria Borkowska, Anna Szumera-Ciećkiewicz, Bartłomiej Szostakowski, Andrzej Pieńkowski, Piotr Lukasz Rutkowski

**Affiliations:** 1 Department of Soft Tissue/Bone Sarcoma and Melanoma, Maria Sklodowska-Curie National Research Institute of Oncology, 02-781 Warsaw, Poland; aneta.borkowska@coi.pl (A.M.B.); bartlomiej.szostakowski@pib-nio.p (B.S.); pienkowski.j.a@onet.eu (A.P.); 2 Department of Pathology and Laboratory Medicine, Maria Sklodowska-Curie National Research Institute of Oncology, 02-781 Warsaw, Poland; anna.szumera-cieckiewicz@pib-nio.pl; 3 Diagnostic Hematology Department, Institute of Hematology and Transfusion Medicine, 02-776 Warsaw, Poland

**Keywords:** giant cell tumor of bone, GCTB, denosumab, RANK, RANKL

## Abstract

**Simple Summary:**

The widely accepted local therapy in extremity giant cell tumor of bone (GCTB) is surgery, in the form of extended intralesional curettage with adequate disease clearance and retention of the limb, wherever possible. Denosumab is a relevant therapy option for advanced GCTB, to benefit tumor response and surgical down-staging. Most GCTB patients with localized disease can be successfully treated with surgical curettage; patients with primary unresectable lesions or metastases may experience long-term clinical and radiological remission and pain control with denosumab treatment, and in this clinical situation, denosumab is currently the treatment of choice.

**Abstract:**

(1) Despite the benign nature of the giant cell tumor of bone (GCTB), it shows a local recurrence rate of up to 50% and a chance of malignant transformation. The widely accepted local therapy in extremity GCTB is surgery, in the form of extended intralesional curettage with adequate disease clearance and retention of the limb, wherever possible. Denosumab, a human monoclonal antibody directed against the RANKL and associated inhibition of the RANKL pathway, is a relevant therapy option for advanced GCTB, to benefit tumor response and surgical down-staging. (2) The literature review of patients with GCTB treated with denosumab is performed via PubMed, using suitable keywords from January 2009 to January 2021. (3) Current indications for denosumab use are not definitively clear and unambiguous. Most GCTB patients with localized disease can be successfully treated with surgical curettage, and the role of denosumab in preoperative therapy in this patient population remains unclear. (4) However, patients with primary unresectable lesions or metastases may experience long-term clinical and radiological remission and pain control with denosumab treatment, and in this clinical situation, denosumab is currently the treatment of choice.

## 1. Pathophysiology and Diagnostics of Giant Cell Tumor of Bone

### 1.1. Bone Microenvironment

**Figure 1:**
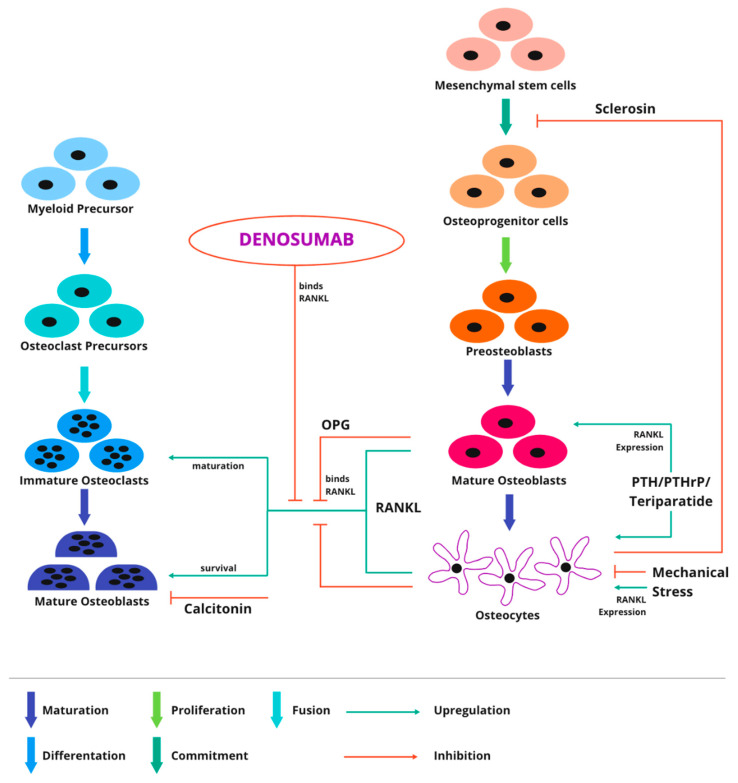
The basic metabolic unit’s physiologic signaling pathway and its precursors. The osteocyte is the main cell responsible for osteological metabolic regulation, as it regulates osteoblasts through sclerostin and controls osteoclasts through RANKL/OPG expression. RANKL increases osteoclastogenesis and osteoclast survival by acting on RANK. RANKL expression and signaling are critical mechanisms for activating osteoclasts and driving subsequent bone remodeling through hydroxyapatite and collagen resorption events. RANKL—nuclear factor-kB receptor activator ligand; RANK—receptor located on osteoclasts; OPG—osteoprotegerin; PTH—parathyroid hormone; PTHrP—parathyroid hormone-related protein.

Bone is a mineralized hydroxyapatite (HA) matrix with many diverse functions, primarily providing structural support to the body and locomotion. Still, it is also a site associated with hematopoiesis, immune cell maturation, and a calcium/phosphorus reservoir. Strong bone vascularization supports the high metabolic turnover of intramedullary hematopoietic stem cells, bone matrix osteocytes, and periosteal progenitor cells [1]. Normal bone mass, architecture, and mineral homeostasis are maintained through continuous renewal based on a dynamic balance between osteoclastic bone resorption and osteoblastic bone formation. Osteoblasts mineralize bone by synthesizing HA, whereas osteocytes provide nutrients and waste removal to mature bone. In both physiological and pathological bone resorption, osteoclasts play a key role. A crucial cytokine that induces osteoclast differentiation and activation is the nuclear factor-kB receptor activator ligand (RANKL). The precursors of remodeling cells are mesenchymal stem cells that differentiate into mature osteoblasts under the influence of cytokines. Osteoblasts form a scaffold of immature bone, which fuses with hydroxyapatite. Under the influence of parathyroid hormone (PTH), osteoblasts produce the receptor-ligand for RANKL, which is important for bone metabolism. The binding of RANKL to the receptor located on osteoclasts (RANK) causes osteoclast differentiation and maturation [2]. During osteogenesis, osteoblasts deposit osteoid and transform into osteocytes, which are deposited in the bone matrix. Osteocytes support the bone matrix by replenishing nutrients and removing. Osteocytes respond primarily to PTH, mechanical stress, and inflammatory cytokines. Osteocytes play a key role in bone metabolism through the upregulating and downregulating factors interacting with osteoblasts and osteoclasts. Osteocytes are unable to divide. They can increase the expression of transmembrane RANKL, thereby regulating osteoclastic activity necessary for bone remodeling. Osteoclasts under the influence of RANKL, macrophage colony-stimulating factor (M-CSF), and other cytokines aggregate to form giant cells with multiple nuclei that eventually mature into osteoclasts. By demineralizing HA through a series of acid pumps and collagen digestion by enzymes, osteoclasts lead to bone resorption. Therefore, osteoclasts are one of the primary cells that induce bone pathology morbidities. Figure 1 shows the physiologic signaling pathway of the basic metabolic unit and precursors and increased osteoclastogenesis and osteoclast survival by RANKL acting on RANK. Due to the important role of osteoclasts in bone disease etiopathogenesis, most osteologic pharmacologic agents target osteoclasts in some way [2].

### 1.2. Giant Cell Tumor of Bone

GCTB (giant cell tumor of bone) is osteolytic and is usually a benign tumor. GCTB of bone accounts for 20% of benign tumors in the adult population but represents less than 5% of all primary bone tumors, with an estimated incidence of 1.3 per million per year [3]. Despite the benign nature of the GCTB, it exhibits a vast range of behaviors, including a local recurrence rate of up to 50%, multicentricity, distant metastatic lesions, invasiveness, and up to 10% chance of malignant transformation [4]. The peak age of occurrence is the third decade of life in a mature skeleton, with a female predominance. GCTB occurs as a primarily solitary tumor at several osseous locations but commonly arises at the meta-epiphyses of long bones. Most lesions develop around the knee region and can grow in the proximal humerus, distal radius bone, spine, and pelvis. Initially, tumor expansion begins from local bone structures, with later possible involvement of surrounding soft tissues [5,6,7].

In less than 10% of cases, GCTB is located in the spine and sacrum. Sacral GCTB accounts for 2–8% of all GCTB cases, making it more frequent than pelvic GCTB [8]. The mobile spine is afflicted in 54.8% of GCTB patients, whereas the sacrum is impacted in 45.2% [9]. As with extremities with GCTB, there is a slight female predominance. Axial location of GCTB is challenging in diagnosis and management with high recurrence rates of up to 41.7% and pulmonary metastasis in a range from 0 to 13.7% [10,11]. Because of the rarity of GCTB located in the spine and sacrum, data come from case reports.

A group of GCTBs are characterized by reactive multinucleated osteoclastic giant cells that express RANKL [4]. In addition to GCTB, giant cell granulomas, aneurysmal bone cysts, and chondroblastomas are also included in this tumor category [12]. Fatigue, muscular pain, arthralgia, extremity pain, and back pain were the most common GCTB patient complaints reported in the literature [7].

#### Histopathology and Genetics

**Figure 2:**
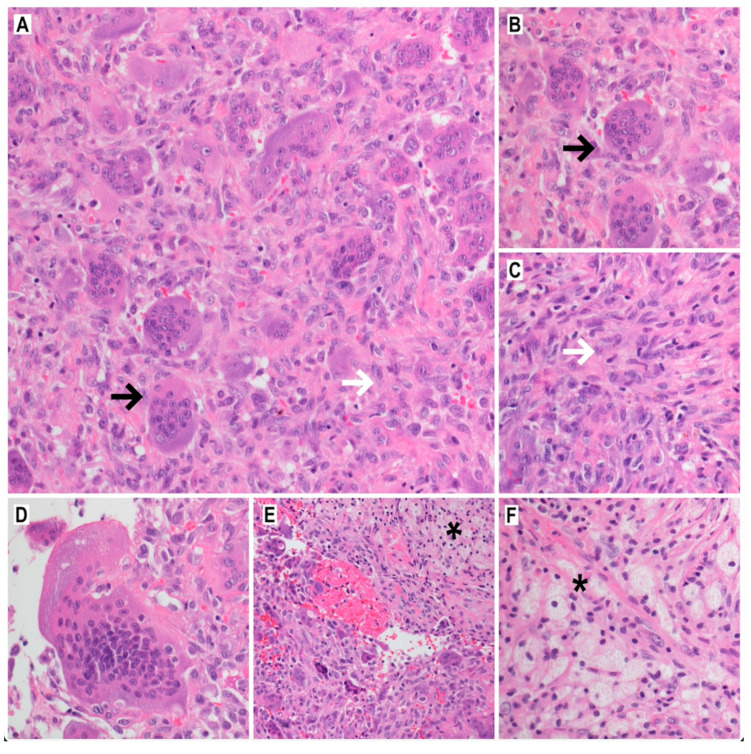
Giant cell tumor of bone: the characteristic mixture of osteoclast-like giant cells (**A**, black arrow, HE, 200×; **B**, black arrow, HE, 400×), and mononuclear neoplastic cells (**A**, white arrow, HE, 200×; **C**, white arrow, HE, 400×); some of the giant-cells have >50 nuclei per cell (**D**, HE, 600×); hemorrhages (**E**, HE, 200×), and field of foamy macrophages (**E**, arteriks, HE, 200×; **F**, arteriks, HE, 400×) are present in majority of cases.

Macroscopically, GCTB comprises highly vascularized collagen bands with hemorrhages, hemosiderin deposits, and foamy macrophages [13]. Histologically, in GCTB are three cell lineages: typical large multinucleated osteoclastic giant cells, spindle-shaped mesenchymal stromal cells, and rounded mononuclear histiocytic or macrophage-like osteoclast precursor cells (Figure 2A–F) [4,5,6]. Neoplastic stromal cells, which are the main neoplastic component of the tumor, have poorly defined cytoplasm with spindle-shaped nuclei (Figure 2C). They exhibit a high expression of RANKL. This and many other endogenous factors are involved in increasing RANKL expression and decreasing osteoprotegerin (OPG) expression. Therefore, these cellular pathways are potential therapeutic targets [14].

Overexpression of RANKL by mononuclear neoplastic stromal cells promotes the recruitment of reactive multinucleated osteoclast-like giant cells capable of lacunar bone resorption via cathepsin K. The cathepsin K is a principal protease exclusively expressed in these osteoclast-like giant cells, according to functional biology. Mononuclear osteoclast precursor cells differentiate into multinucleated osteoclast-like giant cells. Osteoclastogenesis observed in GCTB is primarily due to RANK-RANKL interaction and M-CSF binding [6]. As a result, giant cells present an osteoclasts-like immunophenotype (positive for CD45/LCA, CD68, CD33 and negative for CD14, CD163, HLA-DR) [15]. The giant cells are usually larger than normal osteoclasts and have vesicular nuclei and eosinophilic cytoplasm. Atypical mitotic figures indicate malignant change. Giant cells overexpress the RANK.

**Figure 3:**
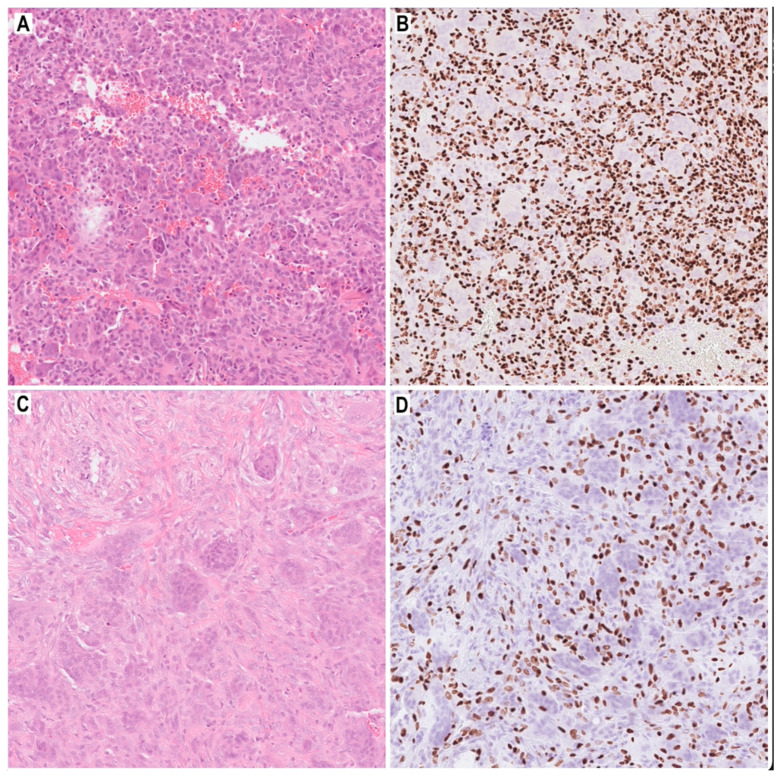
Giant cell tumor of bone: predominant mononuclear cells (**A**, HE, 100×; **B**) with strong nuclear expression of H3.3 p.Gly34Trp (G34W) (**B**, G34W, 100×); the giant cells (**C**, HE, 100×; **D**, G34W, 100×) are negative for H3.3 p.Gly34Trp (G34W) but positive reaction in mononuclear cells are described in over 90% of cases (**D**, G34W, 100×).

Genetic analyses may soon be an important diagnostic factor in predicting the clinical behavior of GCTB. A chromosomal abnormality in the form of telomeric associations is observed in 50–70% of GCTB cases. A structural telomere protective-capping mechanism plays a central role in telomere length maintenance in GCTB. This mechanism expresses telomerase maintenance markers in spindle-shaped mononuclear neoplastic stromal cells and mononuclear rounded osteoclast precursor cells. Genomic analyses identified a driver mutation in the H3F3A gene on histone-3.3 in the neoplastic stromal cells of GCTB, but not in precursor or mature osteoclasts. Additionally, H3F3A driver mutations are specific for GCTB and H3F3B for chondroblastoma, thus distinguishing the two conditions [6,16,17]. The immunohistochemical assessment of H3F3A and H3F3B protein overexpression is an important diagnostic tool in routine histopathological diagnostics (Figure 3). In a study of nine patients with GCTB who underwent curettage after neoadjuvant denosumab therapy, authors evaluated immunohistochemistry markers before and after denosumab therapy. An osteoclast-like giant cells marker NFAT1c, an osteoblast marker RUNX2, and histone H3.3 G34W were examined. Moreover, H3F3A mutation status was performed. Before treatment, tumor cells were NFATc1, RUNX2, and G34W positive, and contained H3F3A mutations. After denosumab therapy, the number of osteoclast-like giant cells was reduced. The neoplastic cells with the H3F3A mutation, on the other hand, survived denosumab treatment and underwent significant histological alterations [18].

In 2.1–6.6% of individuals with advanced or recurrent GCTB, pulmonary metastases with often-latent behavior occur [7,19,20]. Metastatic disease is considered to result from increased bone resorption, by which tumor cells are released for spread to distant sites. This increased bone resorption is caused by the stimulation of osteoblasts by factors secreted from the tumor, which increase the expression of RANKL [21]. Recurrent and metastatic disease is correlated with the increased regulation of epidermal growth factor receptor, a tyrosine kinase expressed by neoplastic stromal cells to enhance osteoclastogenesis, in response to M-CSF [6]. Chromosome aneuploidy and centrosome amplification were associated with recurrence and metastasis in GCTB, which may help determine the malignancy of the disease [22]. Lower expression of the genes that control cell membrane stability, decorin, and lumican has also been associated with metastatic disease and could be used as biomarkers for metastatic and recurrent GCTB [23]. Mutations in H-RAS and TP53, not found in benign GCTB lesions, characterize transformation to malignancy. Evaluation of these mutations may be a significant predictor of disease progression [24]. Despite some hints, histopathological and genetic characteristics of GCTB have not been demonstrated to be clearly predictive of clinical outcomes, such as local progression and the probability of recurrence or metastasis. The advancement of foundational GCTB information could be further analyzed and fused with clinical and radiological aspects to create a multidisciplinary classification of GCTB that can predict clinical behavior and create individual risk profiles.

### 1.3. GCTB Imaging

**Figure 4:**
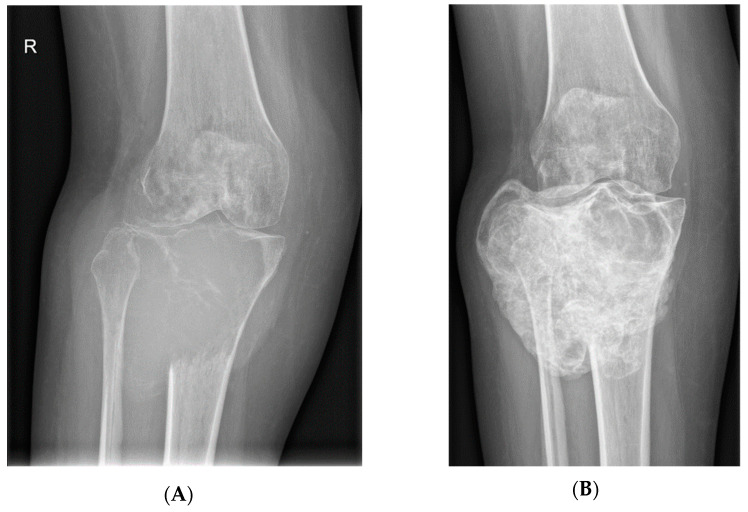

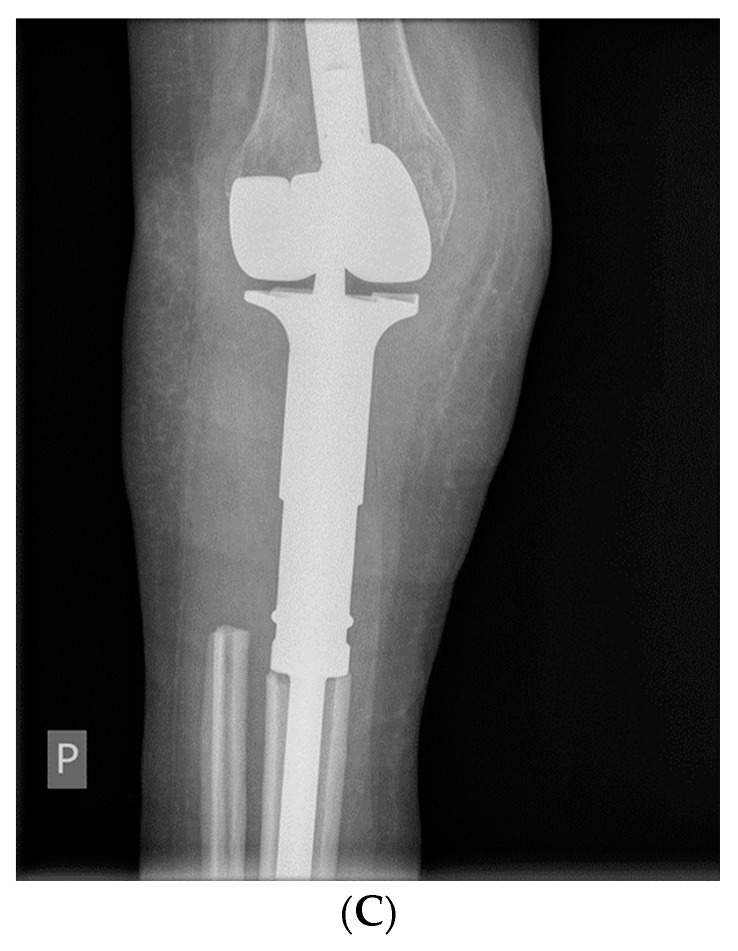
Significant calcification of the locally advanced GCTB of proximal tibia after 8 doses of denosumab (X-rays). Prolonged denosumab treatment can aid resection of the initially inoperable cases. (**A**): Lesion before denosumab therapy, (**B**): after 8 doses of denosumab, (**C**): after the surgery with radical resection and implantation of oncological prosthesis.

**Figure 5:**
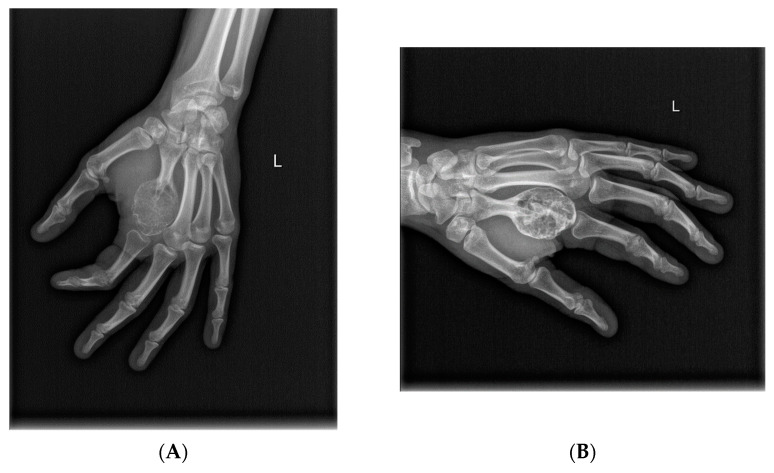

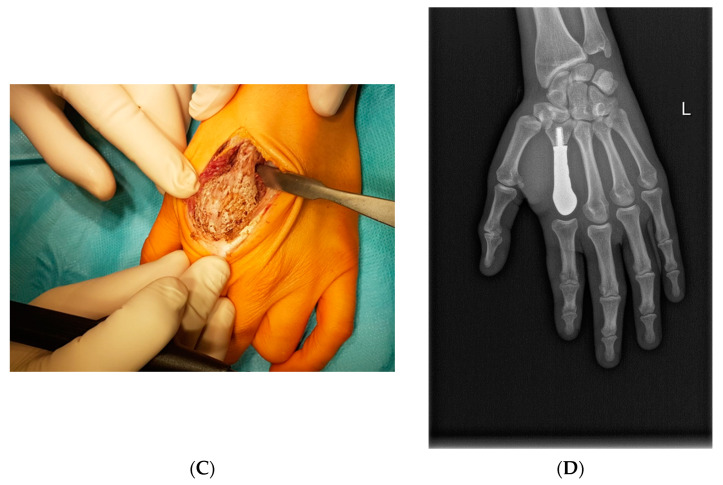
Response to denosumab therapy and radical resection of the GCTB of the second metacarpal followed by reconstruction with a 3D-printed patient-specific implant. (**A**) GCTB of the second metacarpal at the beginning of therapy; (**B**) X-ray taken after 13 doses of denosumab treatment; (**C**) Intraoperative image showing well-calcified tumor after dissection of surrounding soft tissue envelope; (**D**) 3D-printed patient-specific implant.

**Figure 6:**
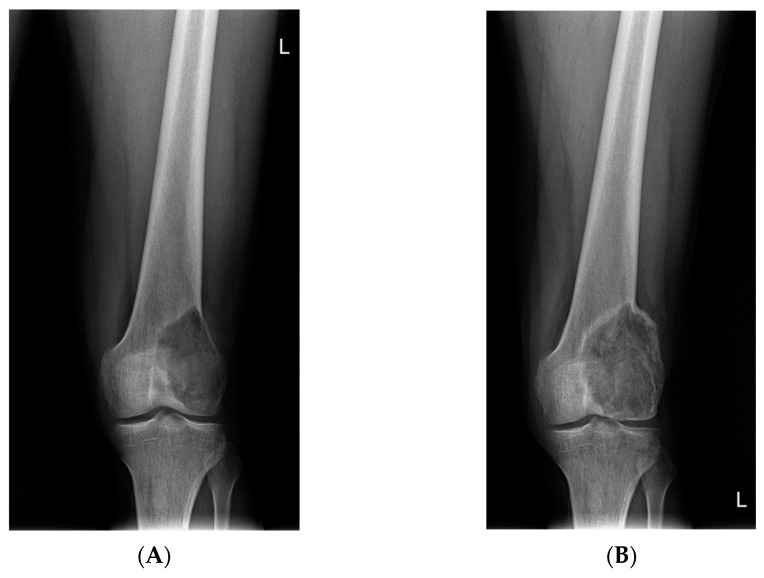

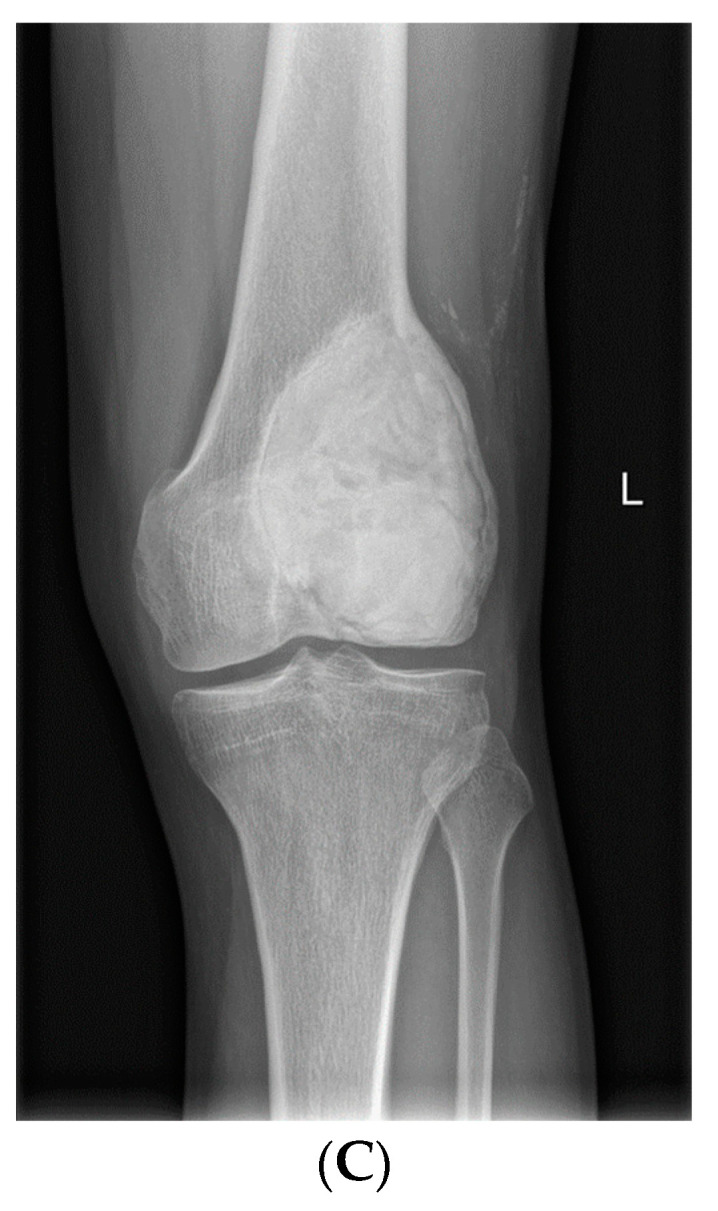
GCTB of the distal femur (X-rays). (**A**) Before denosumab therapy, (**B**) after 6 doses of denosumab, and (**C**) after intralesional curettage and PMMA cementoplasty.

The recommended initial diagnostic in addition to the physical examination is a plain radiograph, for both primary and recurrent GCTB lesions. However, there is currently no consensus on the optimal imaging modalities to monitor patients treated with denosumab. For therapeutic decisions, it is important to identify radiological features of GCTB during denosumab treatment that indicate a positive response to denosumab [25] (Figure 4, Figure 5 and Figure 6). The commonly used score system for GCTB is the Campanacci classification. However, it should not be interpreted definitively as a prognostic factor, because many studies have demonstrated no correlation between the Campanacci grade of the tumor and the risk of local recurrence or metastasis. An important feature indicative of a good response to denosumab treatment on radiographic imaging is osteosclerosis, visible as increased radiopacity in the osteolytic area of the tumor. It often occurs at the periphery of the lesion and is characterized by well-defined marginal sclerosis and neocortex formation [25]. GCTB occurs as an eccentric, lytic lesion with a sharply defined no-sclerotic border on plain radiographs. There may be a large zone of transition with cortical damage and a soft tissue component in more aggressive GCTB. The metaphysis of long bones is the most common location, extending to the epiphysis of the subarticular region. If GCTB responds well to denosumab, a simple X-ray will usually reveal the development of a calcified rim surrounding the tumor and/or a decrease in the size of the lesion.

Computed tomography (CT) scans show tumor matrix and marginal sclerosis. Before intralesional surgery, CT can be conducted to evaluate cortical thinning, pathologic fractures, and fracture consolidation. The CT shows neocortex formation well with a reconstruction of areas of cortical and basal bone destruction, which can be also valuable in surgical planning [25]. Another important application of CT imaging is the detection and monitoring of lung metastases. Pulmonary metastases that respond well to denosumab treatment respond with size reduction or ossification [26].

Magnetic resonance (MRI) imaging is recommended before surgical treatment. MRI is effective for evaluating the response to denosumab treatment, and it is necessary to assess the amount of GCTB within the bone and surrounding soft tissues to establish a surgical strategy. To evaluate the response to denosumab treatment, it is very useful to assess the reduction in the size of the soft-tissue component of GCTB in MRI. Moreover, T1 and T2 signal intensity decreases under good treatment response. The decrease in MR signal intensity may reflect a histological response involving the replacement of multinucleated giant cells and mononuclear cells by fibroblast-like spindle cells, dense fibro-osseous tissue, or woven bone. This response may occur with the low-signal marginal sclerosis and low-signal internal matrix [25,27,28].

PET-CT is valuable in assessing response to treatment. It can assess SUVmax before and after treatment (functional assessment) and monitor the change in tumor size and development of new bone formation on the associated CT image (morphological assessment) [25,29,30,31]. Because SUMmax correlates well with treatment response, an aggressive lesion (e.g., giant cell-rich osteosarcoma) should be histologically excluded if SUWmax is not decreased or increased. PET-CT may also be useful in the evaluation and monitoring of lung metastases [25].

Evaluation of bone mineral density (BMD) as a marker of response to denosumab treatment appears promising. However, there is currently limited evidence. A study involving three patients used dual-energy X-ray absorptiometry (DEXA) to measure BMD during denosumab treatment. In all patients, tumor BMD steadily increased during denosumab treatment. No increase in BMD was observed in control areas (lumbar spine and hip) [32]. Further studies are needed to evaluate the feasibility of using DEXA scanning to decide the optimal duration of treatment and timing of surgery.

## 2. Treatment Overview

### 2.1. Surgical Management of GCTB

The widely accepted and most appropriate local therapy in GCTB of the extremities is extended intralesional curettage, which is based on principles of careful, thorough removal of the tumor tissue by judicious curettage of the tumor bed with various sizes of bone curettes and high-speed burrs. Copious lavage with H_2_O_2_ or 0.9% saline and the use of specific adjuvants such as phenol, liquid nitrogen, argon plasma coagulation or polymethyl methacrylate (PMMA) at the time of the surgery can reduce the risk of local recurrence, destroying microscopic disease left behind curettage [6].

Extended intralesional curettage followed by PMMA cementoplasty, bone substitutes or bone grafting remains the standard treatment solution for locally advanced or recurrent GCTB. The local extent of the tumor or anatomical constraints can sometimes disqualify a patient from this type of therapy.

Uncurettable cases of GCTB when salvage of the joint is not achievable usually qualify for a segmental resection followed by endoprosthetic reconstruction with a modular or patient-specific implant, biologic reconstruction with a bone graft or, in rare cases, amputation of the limb when local conditions, the advancement of the tumor or malignant transformation exclude limb-sparing surgery. For bones that are considered dispensable, such as the proximal part of the fibula, en-bloc resection is an appropriate surgical choice. Lesions may also be considered unresectable if the expected morbidity and mortality rates caused by surgery represent an unreasonable risk. Lesions of the axial skeleton, such as the sacrum, are commonly referred to as unresectable lesions [6].

The rates of local recurrence of GCTB vary widely according to the initial treatment modality used. Disease recurrences are usually observed within 2 years, with a rate of 27–65% for isolated curettage [6]. However, many studies have been conducted regarding possible adjuvant treatments to reduce the risk of local recurrence. The use of high-speed burr, phenol or liquid nitrogen, and polymethyl methacrylate (PMMA) to prolong intraoperative curettage improved local efficacy and reduced the recurrence rate to 12–27% [6,33]. The combination of phenol and PMMA demonstrated minimal difference compared to PMMA alone. Recurrence rates after en-bloc resection are estimated to be 01–12%. In addition, up to 10.5% of recurrent lesions may develop features of malignancy and yield lung metastases [4,34]. Soft tissue extension or location in the sacrum or spine can contribute to the increased chance of local recurrence, which may be explained by the locally aggressive nature of GCTB and technical challenges, incomplete tumor removal, and application of local adjuvants close to neurovascular structures [6,35].

The extent of the soft tissue component, which is likely to improve after neoadjuvant systemic targeted denosumab therapy, determines the feasibility of intralesional surgery. Pathologic fractures are common at presentation (15–20%), but they do not enhance the risk of local recurrence therefore, curettage with adjuvants is a viable treatment option [6].

Because of the complex anatomy and proximity to vital neurovascular structures, adequate disease clearance, whether by intralesional extended curettage or resection, is always a difficulty and is thus linked to a high rate of local recurrence. Surgical methods can be connected with significant morbidity such as neurovascular deficits and injury to the visceral organs. There is an emphasis on developing less morbid therapeutic options that can balance effective local control with minimum treatment-related morbidity. The current management of GCTB is interdisciplinary, requiring input from musculoskeletal oncologists, intervention radiologists, and medical oncologists [36].

### 2.2. Denosumab in Multidisciplinary Medical Management of GCTB

Systemic therapy and irradiation are not considered the standard of care due to the benign nature of GCTB. However, denosumab is a relevant therapy option for advanced GCTB, to beneficial tumor response and surgical down-staging in patients of GCTB, according to clinical trials and case studies, mostly in cases where before, en-bloc resection or even amputation were the only alternatives for unsalvageable cases. Creating an operable situation and obtaining quick local control is critical, and as a result, systemic therapy may eliminate the need for extensive surgery. Denosumab appears to be useful for surgical treatment optimization by suppressing the recruitment of osteoclast-like giant cells by neoplastic stromal cells, avoiding the osteolysis found in GCTB. Furthermore, it can reduce the tumor size and create a calcified rim around the soft tissue component found in mature GCTB, allowing for curettage with local adjuvants or en-bloc excision in previously unresectable GCTB at a later stage [6]. Although uncommon, neoadjuvant denosumab may help downstaging and facilitate en-bloc resection with endoprosthetic replacement in more severe cases, where before amputation was planned. Denosumab has also facilitated surgical treatment for advanced axial GCTB, where surgery was before unfeasible [37].

**Figure 7:**
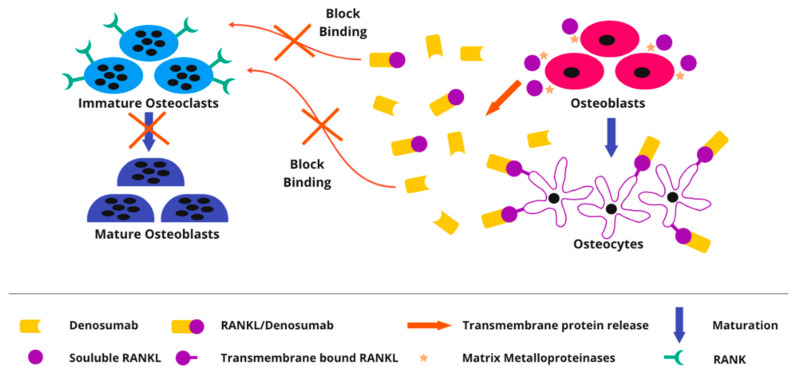
Mechanism of action of a Denosumab, targeting RANKL. RANKL has been demonstrated to exist in a variety of forms. The most physiologically active is the transmembrane version of RANKL on osteocytes. It promotes osteoclast activity upregulation. Metalloproteinases have been demonstrated to release soluble forms of RANKL from the membrane. Denosumab suppresses osteoclastogenesis by binding to RANKL and as a result inhibiting RANKL and preventing RANKL binding to RANK. Denosumab only inhibits osteoclastogenesis, not osteoclast survival. RANKL—nuclear factor-kB receptor activator ligand; RANK—receptor located on osteoclasts.

Denosumab is a human monoclonal antibody directed against the RANKL and associated inhibition of the RANKL pathway. Prevention of RANK activation, therefore inhibits osteoclast activation, mirroring the action of endogenous osteoprotegerin. Thus, it moderates bone resorption (Figure 7) [37,38]. Histopathologically, after denosumab treatment, mononuclear cells and osteoclast-like cells may be no longer detectable [18]. A partial maturation of neoplastic stromal cells towards an osteoblastic phenotype, as well as fibrous and osteoid matrix production, is found after denosumab treatment. Denosumab was approved by the FDA in 2013 for treatment in patients with GCTB who were considered to be either inoperable or surgery would cause unacceptable morbidity or who had metastatic disease. Multiple phase II trials were used to support this approval. Denosumab treatment was found to have a significant clinical, radiological, and histological response in several investigations [39,40,41]. Patients with GCTB after denosumab treatment typically experience pain relief, better function, and mobility [38,42]. Tumor shrinkage, central sclerosis, and bone formation, peripheral bone formation, and complete healing of a pathological fracture were all observed on radiographs after denosumab treatment [43,44,45,46,47]. Although these improvements were observed in almost all studies on denosumab, they did not result in a reduction in local recurrence in the neoadjuvant setting. One probable explanation is that after denosumab treatment, the reactive collagen matrix and osteoid development within the neoplasm make it more difficult to identify tumor from normal tissue [7,48,49,50,51]. As a result, tumor tissue can be left behind during intralesional excision/curettage, leading to recurrence disease following the discontinuation of denosumab treatment [52]. Standard dosing includes administration of 120 mg of denosumab subcutaneously every 4 weeks, with additional doses on days 8 and 15 during the first cycle. Most patients in studies were given denosumab together with calcium and vitamin D supplements. The length of denosumab treatment varied greatly between studies and between participants within the same research, ranging from 4 to 55 months [7].

Trials or case series about GCTB have limitations due to a short follow-up. Moreover, denosumab preoperative therapy is related to the higher likelihood of local recurrence in patients treated with curettage not wide excision. This could be due to the tumor’s thickened bone margin, which trapped tumor cells during curettage. It is considered that the local recurrence was also aided by tumor cells directly forming bone in the margin following denosumab treatment. Denosumab induced a cytostatic rather than a genuine cytotoxic response in neoplastic stromal cells in vitro [3]. The optimal duration, long-term safety, maintenance dose, and optimum indications are still unknown.

### 2.3. Early Evidence of Denosumab Outside GCTB

Denosumab was initially developed by Amgen as a treatment for osteoporosis. Denosumab demonstrated antiresorptive activity and proved relative safety. In the study of 7868 women with osteoporosis, the authors observed a significant reduction in the number of osteoporotic fractures among patients treated with denosumab compared with placebo [53]. A multicenter, randomized study was conducted with 1468 men with non-metastatic prostate cancer treated with androgen deprivation therapy. Patients were randomized to a placebo group and to a denosumab treatment group. According to this research, patients treated with denosumab had an improvement in bone mineral density and a lower incidence of new vertebral fractures [54]. Denosumab was shown to be no worse than zoledronic acid in avoiding or delaying skeletal-related events in individuals with advanced cancer and bone metastases (excluding breast and prostate) or myeloma [55].

#### 2.3.1. Early Evidence in GCTB

The safety and efficacy of denosumab for the treatment of GCTB have been confirmed in several clinical trials. Treatment has been administered both in combination with surgical resection and as a stand-alone approach. In an intermediate analysis of a Phase-2 trial involving 222 GCTB, patients were treated with denosumab (54% women; median age 34 years), whose originally planned surgery was associated with significant functional compromise or morbidity. Forty-eight percent of patients had no surgery at the last follow-up, and 38% underwent a less morbid procedure than initially planned. For 106 patients who did not undergo surgery and continued monthly denosumab intake, the median duration of denosumab intake was 19.5 months. Among 116 patients treated with surgery (median postoperative follow-up 13.0 months), the local recurrence rate was 15%. The authors reported a conclusion that in patients with resectable GCTB, neoadjuvant denosumab therapy resulted in favorable surgical downstaging, including less morbid surgery or no surgery [46].

An open-label Phase-2 study (NCT00396279) conducted from 2006 to 2008, studied 18 patients with recurrent unresectable GCTB, 13 patients with primary unresectable GCTB, and 6 patients with recurrent resectable GCTB. Patients received denosumab until complete tumor resection, disease progression without clinical benefit, or patient decision to discontinue treatment for any reason. Treatment response was assessed histologically or radiographically (when tissue samples were not available). Two patients had insufficient histology or radiology data for assessment. All 20 patients who were evaluated histologically showed elimination of giant cells by 90% or greater from baseline. Ten of the 15 patients evaluated radiologically showed no tumor progression [31].

In the Branstetter et al. study, 20 adult patients with recurrent or unresectable GCTB received denosumab. A decrease of 90% or more in tumor giant cells from the tumor was chosen as the primary histologic efficacy endpoint. In all analyzed samples, a 90% or greater reduction in tumor giant cells was observed. Denosumab treatment also reduced the relative amount of proliferative, dense cellular tumor stromal cells, instead replacing them with nonproliferative, differentiated, densely woven new bone [43].

In a prospective, nonrandomized study reported by Traub et al., 20 patients with resectable GCTB received denosumab for 6 to 11 months before surgery. All patients showed a positive radiographic response with subchondral and cortical bone improvement, allowing tumor resection and joint preservation in 18 cases. All patients experienced a reduction in pain during the first month of treatment. Histologic examination after denosumab administration revealed rarely detectable osteoclast-like giant cells and a marked increase in an osteoid matrix and bone tissue. During a median 30 months follow-up after resection, three patients showed local recurrence. The authors concluded that Denosumab preoperatively provided a favorable and consistent clinical, radiographic, and pathologic response. This facilitates less aggressive surgical treatment, particularly joint preservation. However, denosumab did not reduce the rate of local recurrence of GCTB after resection, which remains a concern [47].

#### 2.3.2. Denosumab Current Clinical Indications in GCTB

Multicenter Phase-2 trials evaluating the safety and efficacy of denosumab therapy in GCTB are currently ongoing. While the usefulness of denosumab in advanced and unresectable tumors is well established, its role in surgically resectable disease is still being debated. Current indications for denosumab therapy include mostly patients with radiologically confirmed GCTB that is recurrent or considered unresectable, where resection would be associated with significant morbidity or mortality. Less morbid surgery offers obvious clinical benefits for the patient, and multiple trials have demonstrated the usefulness of denosumab in reducing surgical procedure morbidity. Currently, the use of neoadjuvant denosumab in operable GCTB is restricted to situations where widespread reactive bone formation and peripheral ossification can facilitate surgery, such as tumors with a large soft tissue component. When preoperative denosumab is given, a planned resection may become less morbid. Denosumab may be considered in the neoadjuvant treatment whenever a segmental resection is thought to be indicated at diagnosis. A six-month preoperative regimen is considered safe and effective. Studies confirmed that denosumab became the optional therapy in selected patients in the multidisciplinary management of GCTB.

A multi-center, open-label Phase-2 study (NCT00680992) was one of the first large investigations evaluating denosumab’s safety profile in GCTB. Patients (*n* = 282) were divided into three groups: with surgically incurable GCTB (cohort 1), with GCTB that can be saved but has a high risk of major morbidity following surgery (cohort 2), and patients who have completed a previous denosumab study in GCTB (cohort 3). The goal of the study was to determine the type, frequency, and severity of adverse events as well as laboratory abnormalities. Time to disease progression in cohort 1 and the proportion of participants who did not have surgery in cohort 2 are two additional outcomes variables [44]. An interim analysis of patients with resectable GCTB treated with denosumab (*n* = 222), indicated a decrease in surgical invasiveness: 86% had clinical benefits, 48% had no surgery, and 38% had less morbid surgery [46]. In the first six months of the study, 92% of patients who got at least one dose of denosumab in cohort 2 did not have surgery [56].

Despite the lack of results from randomized studies comparing surgical treatment of GCTB with and without neoadjuvant denosumab, the neoadjuvant cohorts’ recurrence rates are quite similar to previous data on GCTB treated exclusively surgically. According to the systematic literature analysis, the pooled weighted recurrence rate is 9% [7]. The long-term findings of a Phase-2 study with denosumab in more than 500 patients revealed the following for 157 surgically treated patients: the overall recurrence rate was 27%, 34% for curettage patients, and 12% for those who received resection [41].

Deventer et al. published a single center study of 115 patients with GTCB treated between 2009 and 2019. Mean age of patients was 33.9 years, with predominance of women (59.1%). Most of lesions were located on the distal femur (37.4%) and proximal tibia (24.3%). Lung metastases occurred in four cases. Majority of cases underwent intralesional curettage (91.3%), four patients were treated with a wide resection, and three cases were treated by simple observation. In fourteen of the patients, preoperative denosumab treatment was indicated. Denosumab was applied also preoperatively in 17 cases of local recurrence. In conclusion, preoperative denosumab did not significantly increase the local recurrence [57].

There are also many series and case reports of the denosumab treatment in patients with GCTB. One of the early large reports of the use of denosumab was presented in a series of 35 patients. In 17 patients, surgery following denosumab treatment was performed. Eleven of the patients had wide en-bloc resection with prosthesis implantation in 10 cases and 6 patients with intralesional curettage. Tumor progression was observed in two patients that underwent intralesional curettage without prosthesis implantation. Beyond that, tumor progression was observed during denosumab therapy in two patients after the previous radiotherapy. The one-year progression-free survival (PFS) rate was 92.8% [58]. Another series of 11 patients in a retrospective study of denosumab in unresectable cases of GCTB shows that six doses of preoperative treatment decreased the tumor size and increased density, and improved clinical symptoms [59]. Another case reports three patients with GCTB of the proximal humerus, sacrum, and proximal femur. In all cases, the authors describe safety and great response to denosumab. One of the cases was a previously unresectable lesion in a 27-year-old man, which facilitated the safe use of extended curettage and cementation after 6 months of treatment [60]. The case reported by Park et al. describes the use of denosumab without surgical intervention in a 25-year-old man with a Campanacci grade 3 GCTB on the distal radius bone. At a 3.5-year follow-up and after a 1-year drug-free period, the patient was asymptomatic with no histologic features of tumor recurrence [61]. Karras et al. described a good response to denosumab treatment in a 10-year-old girl with GCTB of the right patella. The patient was previously in a wheelchair. She returned to activity within 4 months of treatment and underwent successful surgical resection 3 months later [62]. Another case report describes a 17-year-old patient with GCTB of the distal femur. Three months after primary surgical resection, the patient developed local recurrence and one month later thorax CT showed bilateral pulmonary nodules metastases. Application of chemotherapy showed no effect, but progression in lungs. After denosumab administration, patient achieved a significant regression at the site of recurrence and pulmonary metastases [63].

There are many case reports of the successful use of denosumab in patients with GCTB of the spine who could not undergo initial intradiscal excision. Denosumab therapy for spinal GCTB reduces tumor stage, surgical difficulties, and neurological impairment progression; nevertheless, it does not result in total GCT cell eradication [64]. Mattei et al. described a case of GCTB of the C2 vertebral body and odontoid process in a 22-year-old female patient. The patient shows complete remission under denosumab treatment and complete radiographic response at 16 months of follow-up [65]. Dubory et al. presented a series of eight patients with GCTB of the spine treated with denosumab. The mean duration of denosumab therapy was 12.9 months. During a follow-up (mean 19.3 months), all patients improved in back pain and neurologic deficits. For patients treated with neoadjuvant denosumab, histologic analysis showed a maximum of 10% alive tumor cells and no giant cells. In CT scans performed after 6 months, a reduction in tumor size was observed, which made surgical interventions safer and less complicated [66].

Denosumab was also applied as a preoperative neoadjuvant to delay or stop the progression of GCTB and to create a sclerotic bone rim. This approach allows for less bone resection and increases the possibility of achieving complete excision with extended curettage, even in previously unresectable lesions [67]. Another example is a case of GCTB in an 84-year-old Japanese man. On magnetic resonance imaging, the tumor measured 94 × 66 × 90 mm and was located in the left iliac bone. Due to the unresectability of the lesion, the patient was treated with denosumab with good response and no side effects [68]. Another case report of a 19-year-old male with GCTB of the sacrum showed successful treatment of neoadjuvant denosumab which allowed for curettage instead of sacrectomy. Moreover, after 10 months of denosumab treatment, a reduction in the size of the metastatic pulmonary nodule was achieved, allowing for curative thoracoscopic surgery [69].

Another example shows that surgeons should be aware that prolonged adjuvant denosumab therapy may increase the difficulty of excision. After 10 cycles of neoadjuvant denosumab therapy, a 51-year-old man with T12 GCTB had his tumor totally excised with a total spondylectomy. Postoperatively, radiographic findings revealed epidural tumor decrease in the spinal canal as well as the creation of a sclerotic rim, where the affected vertebra collapsed. Increased woven bone was found around the resected vertebra’s peripheral lesion, and RANKL-positive stromal cells remained around the woven bone. The tumor margins became unclear as a consequence of these morphological alterations, making excision more challenging. In conclusion, authors suggest that the GCTB stromal cells persisted after long-term denosumab treatment and total surgical excision of primary lesions is the gold-standard treatment, even after denosumab administration [70].

Denosumab can also be used to treat patients with GCTB at the disseminated stage. Miles et al. report the case of a 36-year-old female patient with GCTB of the wrist who developed multiple lung metastases 2 years after resection of the primary lesion. The lung metastases underwent resection. Three years later, unresectable lung dissemination occurred. The patient received treatment with denosumab. A CT scan performed 4 months later showed regression of the lesions. Good response to denosumab persisted for 9 years, with good tolerance [2]. In a study of 381 patients with GCTB, 19 patients developed lung metastases. The kind of surgery and local recurrence are the only significant predictors of lung metastases in GCTB patients. Denosumab does not reduce the likelihood of lung metastases [20].

In some studies, authors observed a significantly higher local recurrence rate in GCTB treated with preoperative denosumab followed by curettage compared with the curettage-only group [48]. The systematic review revealed that preoperative denosumab might increase the risk of local recurrence of GCTB treated with curettage. Other studies showed no difference in local recurrence rate [71]. A definite conclusion about preoperative denosumab on the local recurrence of GCTB treated with curettage has not yet been obtained, because of contradictory results across published studies. Because there are no randomized studies and the existing studies seem to be of poor quality due to indication bias (e.g., denosumab treatment of the most aggressive or unresectable tumors), the available information is limited. Denosumab treatment should be approached with caution until additional conclusive, randomized studies demonstrating a benefit (or lack thereof) have been completed. A summary of main studies on GCTB treated with denosumab are shown in Table S1.

#### 2.3.3. Progression to High-Grade Sarcoma

Concerns have been raised about the possibility of developing progression to high-grade sarcoma while taking denosumab. Malignant GCTB can occur as a high-grade sarcoma within an otherwise GCTB, or as a secondary result of earlier radiation or surgery.

It is unclear whether denosumab causes malignant progression, whether it happens despite systemic treatment as a natural course of GCTB, or if it is due to a biopsy error or a misdiagnosis. Due to the high response rate of denosumab in GCTB, each non-responding patient should have a confirmatory biopsy. The likelihood of malignant transformation of denosumab-treated GCTB into sarcoma deserves further investigation. Some malignant changes, such as tumor progression to osteosarcoma or undifferentiated pleomorphic sarcoma, have been described in the literature. Santosh et al. and Wójcik et al. described the histological resemblance of GCTB treated with denosumab to osteosarcoma, with pseudosarcoma without aggressive features associated with sarcoma, in two separate case series [72,73]. In the multi-center study were four occurrences of sarcomatous transformation (1%), which is consistent with previous findings of the natural course of GCTB [56]. There is still a lot of debate over denosumab’s malignant transformational properties, but accumulating data does not confirm this phenomenon and in the authors’ opinion, the most crucial is the correct diagnosis of GCTB using molecular testing before initiation of denosumab therapy. More research is needed to better understand the safety of denosumab in the treatment of GCTB [72,73,74,75].

#### 2.3.4. Future Considerations and Unknowns

Expanding the indication for intralesional surgery to every individual patient who is considered for surgery would result in a significant improvement in terms of functional outcome and quality of life for patients with GCTB. This can be achieved with neoadjuvant denosumab therapy, which avoids more invasive resections, especially in advanced and axially positioned GCTB. However, there are still certain questions to be answered about denosumab’s efficacy, effectiveness, and safety.

#### 2.3.5. Dosing Regimen and Treatment Duration

Studies to date have used widely varying durations of denosumab treatment and dosing regimen. Most data and the longest observation on denosumab treatment tolerance come from the osteoporosis treatment, where patients received denosumab subcutaneously in 60 mg dose every 6 months. Relevant data on the dosing and duration of denosumab treatment in GCTB come from ongoing phase 2 trials. In most of these studies, treatment consists of giving patients monthly subcutaneous injections of denosumab 120 mg, with saturating doses on days 8 and 15 of the first cycle. Patients are additionally advised to take 500 mg of calcium and 400 international units of vitamin D daily. In ongoing Phase-2 trials, patients are expected to continue treatment with denosumab until complete tumor resection occurs, disease progression occurs without clinical benefit, or the patient wishes to discontinue treatment [31,38,40,46]. The rate of relapse after discontinuation of treatment and whether it is related to the length of treatment is also unknown.

The optimal duration time of denosumab neoadjuvant is still unknown. Some authors recommend a neoadjuvant treatment period of 3–4 months [52,76,77]. This is the time required for a cortical rim to form around the entire tumor and soft tissue component, allowing for the emergence of an operative scenario in previously uncurettable GCTB. According to the authors, this is the appropriate time window for neoadjuvant denosumab therapy followed by intralesional surgery. A gum-like layered substance is observed during surgery after longer denosumab therapy, and as the typical macroscopic appearance of GCTB vanishes, complete tumor removal by intralesional curettage becomes more difficult, with a higher risk of leaving residual disease behind, leading to a higher recurrence risk. When aiming for en-bloc excision from previously unsalvageable GCTB, the effect of prolonged denosumab therapy may be waited upon, to allow for maximum calcification and radical tumor removal for a low probability of recurrence. Further studies are important to develop an optimal dosing regimen and treatment duration.

#### 2.3.6. Side Effects of Denosumab

Denosumab treatment is generally well tolerated with a low incidence of serious side effects. In 10 years, the observation of denosumab treatment in osteoporosis was associated with low rates of adverse events. However, it should be noted that the dose administrated in this indication was lower than the dose administrated in GCTB treatment [53]. In a multi-center study of 282 patients with GCTB treated with denosumab, adverse events did not often occur. Hypophosphatemia in 5% of patients, osteonecrosis of the jaw in 3%, pain in the extremity in 2%, and anemia in 1% were the most common grade 3 or worse adverse effects during the treatment phase. Treatment-related mortality accounted for 2% of all deaths (two of which were considered treatment-related; bone sarcoma and sarcoma) [56]. In another study of 115 patients treated with denosumab, jaw necrosis and polyarthralgia with myalgia occurred in 9.1% of patients [57]. Jaw necrosis was also the main complications (11%) of denosumab treatment of 97 patients in a study by Palmerini et al. Other complications were a mild peripheral neuropathy (11%), skin rash (9%), atypical femoral fracture (4%), and hypophosphatemia (4%). The median treatment duration was 12 months [78]. In a study of 138 GCTB patients treated with denosumab (median treatment duration was 8 months), the treatment was well tolerated. Osteonecrosis of jaw was observed in one patient (0,7%). Grade 3 toxicity was observed in two patients; one patients had hypophosphatemia and another one had hypocalcemia. Grade 2 toxicities was observed in about 10% of patients [79]. In an open-label Phase-2 study of 37 patients with GCTB treated with denosumab, 33 of 37 patients presented adverse events, the most common of which were limb pain, back pain, and headache. Five patients experienced serious adverse events, only one of which was considered likely related to treatment: a non-pregnancy-related grade 3 increase in human chorionic gonadotropin concentration. Importantly, there were no cases of malignant transformation attributed to denosumab therapy [31].

#### 2.3.7. Long-Term Effects, Pregnancy, and Children

Much is still unknown about the long-term effects of denosumab therapy in patients diagnosed with GCTB. Most of the data on the long-term effects of RANKL blockade have been obtained in an older patient population [31]. The long-term effects in the young population stays unclear, including the effects on reproductive performance in patients treated with denosumab. Although pregnancy is an absolute contraindication to denosumab treatment, it is an important consideration as GCTB is commonly diagnosed in women of childbearing age [31,37,47,80].

Denosumab therapy for surgically unsalvageable GCTB may be required as a lifelong treatment, as it is still uncertain what the minimum effective dose and duration or maybe interval treatment of denosumab can safely be given. This includes young female patients with incurable disease who want to get pregnant or patients who are fatigued by the disease’s denosumab adverse effects. Furthermore, when long-term therapy is required, safety profiles and optimal dosage must be established. In the study by Palmerini et al. on 43 patients, prolonged treatment with denosumab in monthly schedule has long-term activity in GCTB and the toxicity profile was mild. However, dose-dependent toxicity was observed (including 6% of osteonecrosis of jaw), which implies strict monitoring of patients in a prolonged therapy. Authors suggested that decreased dose-intensity schedules warrant further studies on unresectable GCTB [81].

Moreover, there is a limited literature considering dosing, timing of treatment, safety, efficacy, and toxicity of denosumab in children. In addition, the pharmacodynamics and pharmacokinetics of denosumab have not been studied in young children. Some cases of administration of denosumab in children for indications such as osteogenesis imperfecta type IV, severe hypercalcemia after stem cell transplantation due to osteopetrosis, and extensive fibrous dysplasia of bone were described. However, data are limited to a small case series in the pediatric population with skeletal immaturity, with no consensus whether denosumab may adversely affect growing skeletons [80]. There is also no clear opinion on treatment options after relapse.

#### 2.3.8. Treatment Discontinuation

Following the discontinuation of denosumab therapy, an ex vivo tissue study revealed that the stromal cells are still alive, with reactive proliferation and upregulation of osteoclastogenesis. In addition, after therapy discontinuation, local growth has been reported in various case studies. After terminating a 2-year denosumab therapy, Matcuk et al. documented a fast and local recurrence of GCTB in a 24-year-old woman. The tumor proved resistant to denosumab in this case, and it had to be amputated. The conclusion was that denosumab might have a rather cytostatic effect than a true cytotoxic ability. The suggested resistant mechanism may be that denosumab may induce RANK overexpression in osteoclast-like giant cells by binding RANKL and then induce RANKL overexpression in stromal cells. The tumor remains to be controlled while on treatment but upon cessation goes into a rapid growth, accompanied by severe osteolysis, because of the cytostatic effect of denosumab [82]. In conclusion, there may be a need for lifelong denosumab treatment or definitive surgery prior to cessation. Other authors suggest that a denosumab-resistant clone may have developed independently. Interestingly, the hormonal changes also may influence GCTB. Some authors speculate that estrogen or progesterone receptors may clarify the relationship between risk of progression or recurrence of GCTB during pregnancy [83,84,85].

#### 2.3.9. New Targets for Systemic Therapy and Biomarkers

Since RANKL inhibition only has an indirect effect on GCTB, the neoplastic stromal cells are not directly targeted. To turn systemic therapy into a definite therapy for GCTB, targets for systemic therapy that specifically target neoplastic stromal cells should be established. New targets for systemic therapy may be investigated in the future based on new findings in functional biology and genetics of GCTB. Wnt/b-catenin and recombinant human bone morphogenetic protein-2 are two pathways that modulate neoplastic stromal cells’ osteoclast-inducing activity and could be used as therapeutic targets for direct antitumor therapy. CD33+ is a distinguishing feature of multinucleated giant cells with an osteoclast-like phenotype that can be targeted with gemtuzumab, an anti-CD33+ antibody that has already been used to treat acute myeloid leukemia, and could be a new therapeutic target for GCTB [15]. TNF-alpha, interleukin-6, tumor growth factor-beta, B-cell activating factor, nerve growth factor, insulin-like growth factor (IGF)-I, and IGF-II are all RANKL-independent mechanisms of osteoclastogenesis that, while less potent than RANK/RANKL pathway, may serve as alternative therapeutic targets [6,14]. Biomarkers to predict the risk of relapse are unknown.

## 3. Conclusions

Denosumab is a relatively new, very promising targeted therapy option for patients with GCTB. Several series of GCTB patients treated in routine practice confirmed observations from clinical trials that denosumab is a gold standard in unresectable/metastatic disease and it can be considered in a neoadjuvant setting, with excellent efficacy and tolerability.

However, the current indications for its use are not definitively clear. Most local GCTB patients can be successfully treated with surgical curettage, and the role of denosumab in this patient population is not fully understood, especially in terms of the length of preoperative therapy. Nevertheless, denosumab has a role as an neoadjuvant (induction) therapy in borderline resectable cases, where partial remission of the tumor allows for limb-sparing surgery or facilitates radical operation, especially when en-bloc resection is planned (in these cases, denosumab should be used to maximal effect with full calcification of the tumor penetrating to the soft tissues).

There is no doubt, however, that patients with primary unresectable lesions or metastases may experience clinical and radiological remission and pain control with denosumab treatment. However, there are no clear guidelines as to the details of such a treatment that will most effectively reduce the risk of relapse or progression. Moreover, the schedule of denosumab maintenance dosing and its long-term effects remain undefined, especially in the young population. The potential risks of rapid relapse or transformation of the sarcoma are not fully understood. These issues should be taken into consideration and discussed individually with the GCTB patient during treatment planning and should be the subject of further analysis. The present review of existing experience with denosumab indicates that such therapy should be planned in a multidisciplinary team in experienced reference sarcoma centers.

## Supplementary Materials

The following supporting information can be downloaded at: https://www.mdpi.com/article/10.3390/cancers14092290/s1, Table S1: A summary of GCTB studies.
