# Supplementary material for: Denosumab in Giant Cell Tumor of Bone: Multidisciplinary Medical Management Based on Pathophysiological Mechanisms and Real-World Evidence
Source: Cancers (Basel). 2022 May 4;14(9):2290. doi: 10.3390/cancers14092290 (PMC9100084; doi:10.3390/cancers14092290)
Supplement: Supplementary file 1 [file cancers-14-02290-s001.zip › cancers-1675066-supplementary.pdf]

Table S1. A summary of GCTB studies.

	Number of patients	Year	pre-surgery treatment time in months	post-surgery treatment time in months	Follow-up in months	local recurrence (%)
Rutkowski P et al. [46]	222	2015	6	8	13	13
Traub F et al. [47]	20	2016	>6	no	30	30
Agarwal MG et al. [50]	52	2018	6	no	27	27
Rutkowski P et al. [79]	89	2018	6	6	23	23
Chawla S et al. [41]	157	2019	>6	6	58	58
Puri et al. [51]	44	2019	5	no	34	34
Deventer et al. [57]	33	2022	4	no	65	28,6<br>33,3*

*in cases with previous local recurrence
